# High genetic risk score is associated with early disease onset, damage accrual and decreased survival in systemic lupus erythematosus

**DOI:** 10.1136/annrheumdis-2019-216227

**Published:** 2019-12-11

**Authors:** Sarah Reid, Andrei Alexsson, Martina Frodlund, David Morris, Johanna K Sandling, Karin Bolin, Elisabet Svenungsson, Andreas Jönsen, Christine Bengtsson, Iva Gunnarsson, Vera Illescas Rodriguez, Anders Bengtsson, Sabine Arve, Solbritt Rantapää-Dahlqvist, Maija-Leena Eloranta, Ann-Christine Syvänen, Christopher Sjöwall, Timothy James Vyse, Lars Rönnblom, Dag Leonard

**Affiliations:** 1 Rheumatology and Science for Life Laboratory, Department of Medical Sciences, Uppsala University, Uppsala, Sweden; 2 Rheumatology/Division of Neuro and Inflammation Sciences, Department of Clinical and Experimental Medicine, Linköping University, Linkoping, Sweden; 3 Department of Medical and Molecular Genetics, King’s College London, London, UK; 4 Rheumatology Unit, Department of Medicine, Karolinska Institutet, Karolinska University Hospital, Stockholm, Sweden; 5 Rheumatology, Department of Clinical Sciences, Lund University, Lund, Sweden; 6 Department of Public Health and Clinical Medicine/Rheumatology, Umeå University, Umeå, Sweden; 7 Molecular Medicine and Science for Life Laboratory, Department of Medical Sciences, Uppsala University, Uppsala, Sweden

**Keywords:** antiphospholipid syndrome, cardiovascular disease, gene polymorphism, lupus nephritis, systemic lupus erythematosus

## Abstract

**Objectives:**

To investigate associations between a high genetic disease risk and disease severity in patients with systemic lupus erythematosus (SLE).

**Methods:**

Patients with SLE (n=1001, discovery cohort and n=5524, replication cohort) and healthy controls (n=2802 and n=9859) were genotyped using a 200K Immunochip single nucleotide polymorphism array. A genetic risk score (GRS) was assigned to each individual based on 57 SLE risk loci.

**Results:**

SLE was more prevalent in the high, compared with the low, GRS-quartile (OR 12.32 (9.53 to 15.71), p=7.9×10^–86^ and OR 7.48 (6.73 to 8.32), p=2.2×10^–304^ for the discovery and the replication cohorts, respectively). In the discovery cohort, patients in the high GRS-quartile had a 6-year earlier mean disease onset (HR 1.47 (1.22 to 1.75), p=4.3×10^–5^), displayed higher prevalence of damage accrual (OR 1.47 (1.06 to 2.04), p=2.0×10^–2^), renal disorder (OR 2.22 (1.50 to 3.27), p=5.9×10^–5^), anti-dsDNA (OR 1.83 (1.19 to 2.81), p=6.1×10^–3^), end-stage renal disease (ESRD) (OR 5.58 (1.50 to 20.79), p=1.0×10^–2^), proliferative nephritis (OR 2.42 (1.30 to 4.49), p=5.1×10^–3^), anti-cardiolipin-IgG (OR 1.89 (1.13 to 3.18), p=1.6×10^–2^), anti-β_2_-glycoprotein-I-IgG (OR 2.29 (1.29 to 4.06), p=4.8×10^–3^) and positive lupus anticoagulant test (OR 2.12 (1.16 to 3.89), p=1.5×10^–2^) compared with patients in the low GRS-quartile. Survival analysis showed earlier onset of the first organ damage (HR 1.51 (1.04 to 2.25), p=3.7×10^–2^), first cardiovascular event (HR 1.65 (1.03 to 2.64), p=2.6×10^–2^), nephritis (HR 2.53 (1.72 to 3.71), p=9.6×10^–7^), ESRD (HR 6.78 (1.78 to 26.86), p=6.5×10^–3^) and decreased overall survival (HR 1.83 (1.02 to 3.30), p=4.3×10^–2^) in high to low quartile comparison.

**Conclusions:**

A high GRS is associated with increased risk of organ damage, renal dysfunction and all-cause mortality. Our results indicate that genetic profiling may be useful for predicting outcomes in patients with SLE.

**Key messages**

**What is already known about this subject?**

- The field of genetics has been revolutionised by genome-wide association studies, with over 100 genetic loci associated with systemic lupus erythematosus (SLE) discovered.
- Genetic risk scores have shown promise for understanding the polygenic contribution to many complex diseases but have been scarcely investigated in SLE.

**What does this study add?**

- In the present study, we demonstrate that a high genetic risk is associated with an early onset of SLE, increased organ damage, cardiovascular disease and end-stage renal disease, as well as impaired survival.

**How might this impact on clinical practice or future developments?**

- Our results suggest that genetic profiling of patients with SLE may be useful for predicting outcome of the disease.

## Introduction

Systemic lupus erythematosus (SLE) is a chronic disease characterised by loss of tolerance to self-antigens, formation of immune complexes and an activated type I interferon system.^1–3^ Despite improved prognosis, the mortality rate stills exceeds that of the general population.^4^ Due to active inflammation, prolonged corticosteroid use, comorbidities and factors unrelated to SLE, organ damage accumulates in the majority of patients over time,^1 5 6^ with cardiovascular disease and renal failure being strong risk factors for premature mortality.^4 7–9^

Familial aggregation and twin studies provide compelling evidence of genetic predisposition in SLE, with a more than 10-fold higher concordance rate for monozygotic than for dizygotic twins.^10 11^ The genetic aetiology is complex, with single nucleotide polymorphisms (SNPs) at more than 100 genetic loci associated with SLE identified at genome-wide significance.^1 12–16^ While susceptibility to SLE appears to increase with the number of these risk loci,^13^ specific disease manifestations may be associated with a subset of polymorphisms. For example, variants of Signal Transducer and Activator of Transcription 4 (*STAT4*), have displayed association with nephritis, ischaemic stroke, severe renal insufficiency and a younger age at disease onset^17–20^ as well as an increased overall risk of organ damage.^21^ For the majority of SLE susceptibility loci however, no links to specific disease subphenotypes have been demonstrated.

Comprehension of the genetic contribution to permanent organ damage is important for understanding the pathogenesis of SLE. Additionally, prediction of disease outcome is essential for optimising monitoring and treatment strategies, to reduce both unnecessary side-effects and long-term disease complications. Genetic risk scores (GRSs) have been applied in several fields of medicine, and studies have demonstrated their ability to predict matters like cardiovascular disease, prostate cancer risk and body mass index scores.^22–24^ In SLE, few studies have assessed the relationship between the cumulative genetic risk and disease subphenotypes,^25–28^ and the association between the polygenic risk and disease severity is unknown. In this study, we examined the relationship between a high GRS and clinical manifestations associated with more severe SLE phenotypes, including organ damage, defined by the Systemic Lupus Collaborating Clinics (SLICC)/American College of Rheumatology (ACR) Damage Index (SDI),^29^ cardiovascular events (CVE) and end-stage renal disease (ESRD).

## Patients, healthy individuals and methods

### Patients and healthy controls

The discovery cohort included 1001 patients from the University clinics in Uppsala, Linköping, Karolinska Institute (Stockholm), Lund, and from the four northern-most counties in Sweden. All subjects fulfilled ≥4 ACR-82 classification criteria for SLE and were of European descent.^30^ Clinical data were collected from the patients’ medical files, including SDI scores,^29^ the ACR-82 classification criteria, clinical antiphospholipid syndrome (APS) diagnosis, glomerular filtration rate, chronic kidney disease (CKD) stages, ESRD, renal biopsy data and CVE, defined as myocardial infarction, ischaemic cerebrovascular disease or venous thromboembolism (VTE). For definitions, see online supplementary file 1. Patient characteristics are summarised in table 1. For prevalences of SDI scores per organ domain, see online supplementary table 1. Control individuals were healthy blood donors from Uppsala (Uppsala Bioresource) and Lund or population based controls from Stockholm and the four northernmost counties of Sweden. The replication cohort included 5524 patients with SLE and 9859 healthy controls of European ancestry, defined by principal component analysis, described in Langefeld *et al*.^1^

10.1136/annrheumdis-2019-216227.supp1 Supplementary data

10.1136/annrheumdis-2019-216227.supp2 Supplementary data

**Table 1:** Prevalence of clinical manifestations and serology vs associations with the genetic risk score in the Discovery cohort

	n (%)	GRS, high vs low quartiles		GRS, continuous	
		OR (95 % CI)*	P value†	OR (95 % CI)‡	P value†
Deceased at follow-up	99 (10)	1.79 (0.93 to 3.46)	8.0×10^–2^	**1.30 (1.07 to** **1.59**)	**9.4×10^–3^**
Male gender	132 (13)	1.27 (0.77 to 2.12)	3.4×10^–1^	1.07 (0.91 to 1.24)	4.2×10^–1^
SDI scores^29^		**1.47 (1.06 to** **2.04**)	**2.0×10^–2^**	**1.13 (1.03 to** **1.24**)	**1.4×10^–2^**
SLE criteria, ACR-82^30^					
Malar rash	565 (56)	0.88 (0.61 to 1.26)	5.4×10^–1^	0.94 (0.85 to 1.05)	2.6×10^–1^
Discoid rash	236 (24)	0.85 (0.56 to 1.30)	4.7×10^–1^	0.94 (0.83 to 1.07)	3.4×10^–1^
Photosensitivity	680 (68)	0.75 (0.51 to 1.09)	1.2×10^–1^	**0.88 (0.79 to** **0.99**)	**2.6×10^–2^**
Oral ulcers	249 (25)	1.07 (0.71 to 1.62)	8.5×10^–1^	1.02 (0.91 to 1.15)	7.0×10^–1^
Arthritis	800 (80)	0.74 (0.47 to 1.17)	2.0×10^–1^	0.91 (0.80 to 1.04)	1.5×10^–1^
Serositis	447 (45)	0.95 (0.66 to 1.36)	8.2×10^–1^	0.95 (0.86 to 1.06)	3.6×10^–1^
Renal disorder	342 (34)	**2.22 (1.50 to** **3.27**)	**5.9×10^–5^**	**1.29 (1.16 to** **1.44**)	**7.0×10^–6^**
Neurological disorder	105 (10)	1.12 (0.77 to 1.62)	5.6×10^–1^	1.09 (0.92 to 1.29)	3.3×10^–1^
Haematological disorder	616 (62)	1.04 (0.87 to 1.25)	6.5×10^–1^	1.05 (0.94 to 1.17)	3.7×10^–1^
Immunological disorder	686 (69)	**2.03 (1.38 to** **2.98**)	**3.6×10^–4^**	**1.29 (1.15 to** **1.45**)	**1.6×10^–5^**
dsDNA antibodies	477 (62)	**1.83 (1.19 to** **2.81**)	**6.1×10^–3^**	**1.31 (1.15 to** **1.50**)	**4.2×10^–5^**
Sm antibodies	95 (13)	1.24 (0.65 to 2.37)	5.2×10^–1^	1.10 (0.90 to 1.33)	3.5×10^–1^
ANA	970 (98)	2.29 (0.59 to 8.89)	2.3×10^–1^	1.37 (0.91 to 2.07)	1.4×10^–1^
Renal biopsy data^47^					
WHO Class I-II	32 (14)	1.67 (0.61 to 4.60)	3.2×10^–1^	1.17 (0.86 to 1.59)	3.3×10^–1^
WHO Class III-IV	133 (60)	**2.42 (1.30 to** **4.49**)	**5.1×10^–3^**	**1.36 (1.14 to** **1.62**)	**7.5×10^–4^**
WHO Class V	31 (14)	1.88 (0.70 to 5.10)	2.1×10^–1^	1.10 (0.80 to 1.51)	5.6×10^–1^
Other§	20 (9)	0.95 (0.29 to 3.13)	9.5×10^–1^	1.01 (0.68 to 1.50)	9.5×10^–1^
CKD stages^48^		**2.16 (1.31 to** **3.56**)	**2.6×10^–3^**	**1.26 (1.09 to** **1.47**)	**2.4×10^–3^**
ESRD	24 (2)	**5.58 (1.50 to** **20.79**)	**1.0×10^–2^**	**1.65 (1.18 to** **2.32**)	**3.6×10^–3^**
Antiphospholipid antibodies					
Any aPL	257 (38)	**1.84 (1.16 to** **2.9**)	**9.4×10^–3^**	**1.15 (1.00 to** **1.32**)	**4.9×10^–2^**
Triple positive aPLs¶	119 (20)	**2.27 (1.02 to** **5.09**)	**4.6×10^–2^**	**1.30 (1.02 to** **1.66**)	**3.2×10^–2^**
LA	121 (22)	**2.12 (1.16 to** **3.89**)	**1.5×10^–2^**	**1.21 (1.02 to** **1.45**)	**3.3×10^–2^**
aCL-IgG	181 (27)	**1.89 (1.13 to** **3.18**)	**1.6×10^–2^**	1.14 (0.98 to 1.32)	9.1×10^–2^
aCL-IgM	69 (13)	1.07 (0.5 to 2.29)	8.6×10^–1^	1.13 (0.91 to 1.41)	2.7×10^–1^
aβ_2_GP-I-IgG	118 (18)	**2.29 (1.29 to** **4.06**)	**4.8×10^–3^**	**1.32 (1.11 to** **1.58**)	**2.1×10^–3^**
aβ_2_GP-I-IgM	19 (11)	1.01 (0.98 to 1.05)	4.9×10^–1^	0.91 (0.61 to 1.35)	6.3×10^–1^
Clinical APS	132 (19)	1.35 (0.78 to 2.33)	2.8×10^–1^	1.13 (0.96 to 1.34)	1.4×10^–1^

Values in bold indicate p<0.05.

*OR for the high compared to the low GRS-quartile.

†Unadjusted.

‡OR for every increase of one point in the GRS (eg, from 6.5 to 7.5).

§Patients with biopsies displaying signs of nephritis but not meeting the criteria for any of the above classes^32^ were classified as other.

¶Triple positivity for aPLs was defined as having positive tests for aCL (IgG or IgM) and aß2GP-I (IgG or IgM) and LA.

aCL, anticardiolipin; ACR, American College of Rheumatology; aβ_2_GP-I, anti-β_2_ Glycoprotein-I;aPL, anti-phospholipid antibody; APS, antiphospholipid syndrome; CKD, chronic kidney disease; ESRD, end-stage renal disease; GRS, genetic risk score; LA, lupus anticoagulant; SDI, SLICC Damage Index; SLE, systemic lupus erythematosus; SLICC, Systemic Lupus Collaborating Clinics.

### Genotyping and construction of the genetic risk score

Genotyping of the discovery cohort was performed using the Illumina 200K Immunochip SNP array by the SNP&SEQ Technology platform at Science for Life Laboratory in Uppsala, Sweden. For quality control (QC) procedures, see online supplementary file.

Cumulative GRSs were assigned to each individual based on SNPs with previous association with SLE at genome wide significance in the European population from the publication by Chen *et al*.^13^ The inclusion criteria (see online supplementary file 1) allowed for inclusion of 57 SNPs (online supplementary table 2). For each SNP, the natural logarithm of the OR for SLE susceptibility based on comparisons between the 1001 patients and 2802 controls in the discovery cohort was multiplied by the number of risk alleles in each individual. The sum of all products for each patient was defined as the GRS. In addition, a risk allele count (RAC) of the 57 SNPs in each individual was performed by adding the total number of risk alleles. Finally, an HLA-GRS was constructed, see online supplementary file 1 and online supplementary table 3.

Individuals in the replication cohort were independently genotyped using the Illumina 200K Immunochip SNP array, available at https://www.ebi.ac.uk/gwas/. A RAC and GRS was assigned to each patient and control using the same 57 SNPs and OR as in the discovery cohort analysis, see online supplementary table 2. Individuals included in the discovery cohort analysis or with <100% genotype success rate of the 57 SNPs were excluded from the replication cohort (pi HAT >0.9). For genotyping and QC procedures of the replication cohort, see Langefeld *et al.* 1

### Statistical analysis

We used ordinal or logistic regression to assess differences in prevalences between groups. Age was included as a covariate in all analyses, and significant results were subsequently analysed in a second model, with the age at SLE diagnosis as an additional covariate. The generalised Wilcoxon test was employed to assess differences in survival. For more information on statistical analysis, see online supplementary file. Statistical analyses were performed using R.^31^ Unadjusted p<0.05 were considered statistically significant.

## Results

### Genetic characteristics of patients and healthy individuals

**Figure 1:**
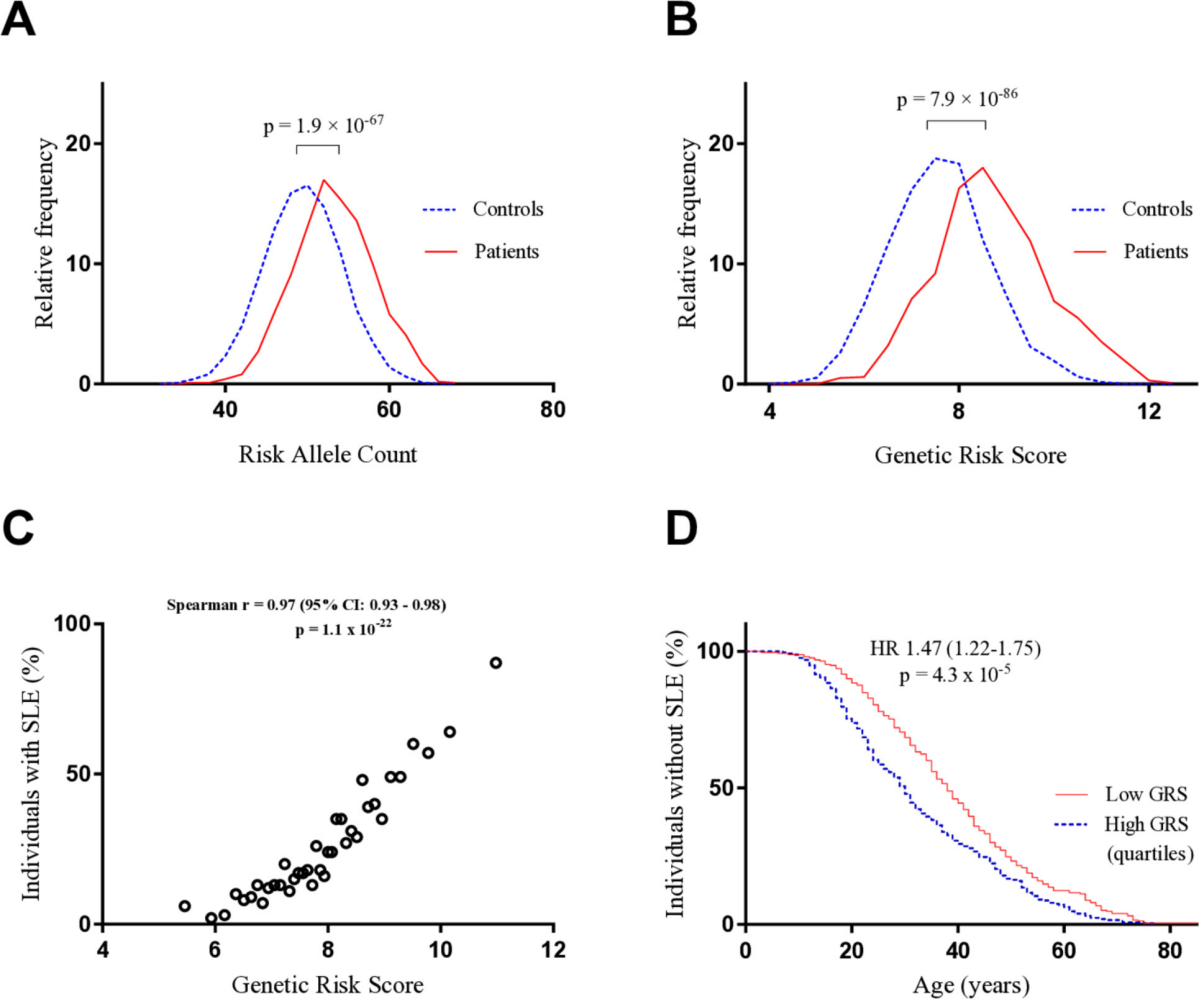
Cumulative genetic risk and SLE development. (A) The distribution of the RAC in the patients (n=1001) and healthy controls (n=2802). (B) The distribution of the weighted GRS in the same individuals. (C) The patients and healthy controls were ordered according to their GRSs and divided into 38 groups, each including 100 individuals (with exception of the first group, which consisted of 103 individuals). The SLE prevalence of each group was plotted against its mean GRS. (D) The survival until SLE onset was analysed for patients with a GRS in the extreme quartiles (n=500). GRS, genetic risk score; RAC, risk allele count; SLE, systemic lupus erythematosus.

Initially, we performed a RAC in each individual in the discovery cohort and as can be seen in figure 1A, the RAC followed a Gaussian distribution, with higher mean scores in patients than in healthy controls (mean (SD) 52.71 (4.81) compared with 48.95 (4.71)). The prevalence of SLE was higher in individuals with a RAC in the highest, compared with the lowest, quartile (OR 7.81 (6.19–9.85), p=1.9×10^–67^). To test whether the difference between groups would increase when considering the contribution to SLE by each SNP, a weighted GRS was constructed. Similar to the RAC, the GRS followed a Gaussian distribution with higher mean scores in patients than in controls (mean (SD) 8.52 (1.20) compared with 7.45 (1.20)) (figure 1B). In the discovery cohort, the probability that an individual had SLE increased with increasing GRS (figure 1C) and was significantly higher in the highest, compared with the lowest, GRS-quartile (OR 12.32 (9.53 to 15.71), p=7.9×10^–86^). Moreover, patients with a GRS in the high quartile received their SLE diagnosis significantly earlier in life, with a mean age at SLE onset in the high and low quartiles of 33 and 39 years, respectively (figure 1D).

**Figure 2:**
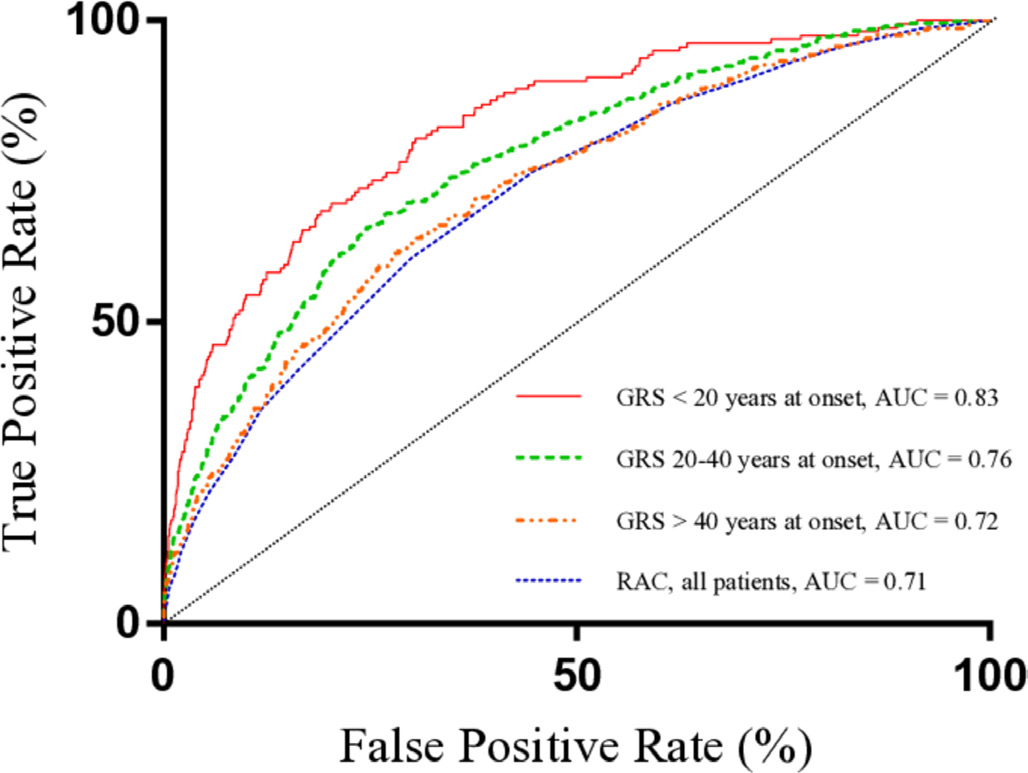
Prediction accuracy of the weighted GRS depending on age at SLE onset. ROC curve analysis was used to assess the prediction ability of the GRS in patients aged below 20 (n=158), 20–40 (n=475) and >40 (n=368) years at SLE diagnosis. The prediction accuracy of the unweighted RAC is shown in the same figure. AUC, area under the ROC curve; GRS, genetic risk score; RAC, risk allele count; ROC, receiver operating characteristic; SLE, systemic lupus erythematosus.

We subsequently employed receiver operating characteristic (ROC) curve analysis to compare prediction accuracies of the scores. The GRS was significantly better than the RAC at discriminating between patients and controls (area under the ROC curve (AUC) 0.78 compared with 0.71, p_comparison_=1.4×10^–14^). In addition, the prediction accuracy of the GRS was higher in patients<20 years at SLE onset (p=3.0×10^–3^ compared with patients aged 20–40 years at onset, p=2.35×10^–6^ compared with patients aged >40 years at onset) (figure 2).

### Replication cohort validation

The RAC and the GRS were validated using genetic data from a replication cohort including more than 15 000 patients and controls. Results show a higher probability of SLE in the high, compared with the low, quartile both for the RAC (OR 5.84 (5.23 to 6.53), p=5.47×10^–213^) and the GRS (OR 7.48 (6.73 to 8.32), p=2.2×10^–304^). Online supplementary figure 1A illustrates the correlation between GRS and prevalence of SLE in this cohort. In the replication cohort, ROC curve analysis showed AUCs of 0.68 and 0.71 for the RAC and the GRS, respectively (p_comparison_=2.2×10^–16^) (online supplementary figure 1B).

10.1136/annrheumdis-2019-216227.supp3 Supplementary data

### Genetic risk score associations

Because the GRS was superior to the RAC in discriminating between patients and controls, subsequent analyses focused on this score. The GRS was analysed as a continuous variable in all regression analyses, with table 1 presenting ORs for a one-unit increase in the GRS. To simplify the interpretation of ORs, we also compared patients with a GRS in the extreme quartiles. There was no significant difference in SLE disease duration between the high and low GRS-quartiles (OR 1.00 (0.99 to 1.02), p=6.7×10^–1^).

**Figure 3:**
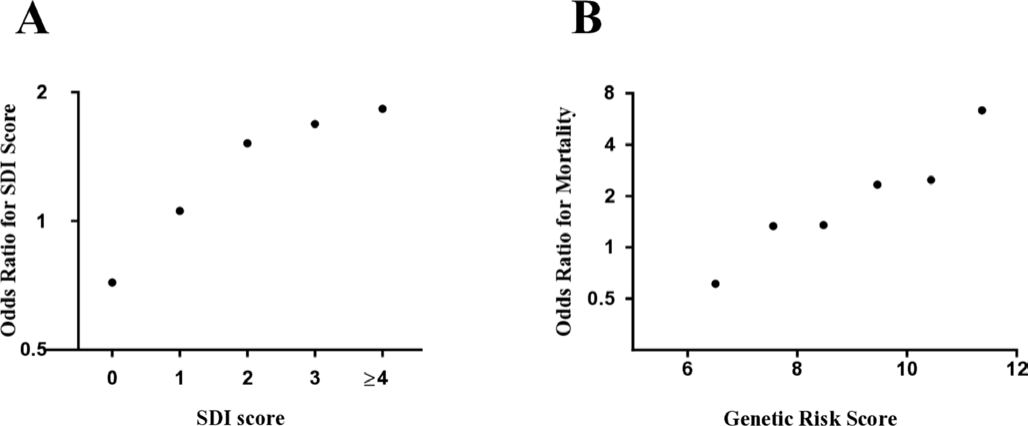
Association of high GRS with organ damage and overall mortality. (A) In five separate logistic regression models, the probability of having 0 vs >0, or 1/2/3/≥4 vs 0, points on the SLICC SDI was calculated for patients with a GRS in the high, compared with the low, quartile. Age was included as a covariate in the analyses. (B) Using the same statistical model and covariate as in A, the OR for mortality compared with patients with a GRS<7 was plotted for patients with a GRS of 7–8, 8–9, 9–10, 10–11 and >11. Patients with a GRS<7 were compared with patients with a GRS>7. GRS, genetic risk score, SDI, SLICC Damage Index; SLICC, Systemic Lupus Collaborating Clinics.

The prevalence of organ damage, as defined by the SDI, increased with increasing GRS (p=1.4×10^–2^). Figure 3A illustrates the probability of having each individual SDI score for patients in the high, compared with the low, GRS-quartile, with 52%, 67% and 83% higher odds of having 2, 3 or ≥4 points on the index, respectively. In the survival analyses, the high and low GRS-quartiles were compared. The mean survival until the first organ damage was decreased in the high quartile (p=3.7×10^–2^), with affected individuals acquiring their first damage at a mean age of 43 years, compared with 51 years in the low GRS-quartile (table 2).

**Table 2:** Survival comparisons based on patients with a GRS in the extreme quartiles in the Discovery cohort

	N patients		Mean age at event* (mean survival†)		HR (95% CI)	P value‡
	Affected	Unaffected	High quartile	Low quartile		
First SDI score	124	92	43 (51)	51 (59)	**1.51 (1.04 to** **2.25**)	**3.7×10^–2^**
First CVE	114	308	45 (64)	51 (70)	**1.65 (1.03 to** **2.64**)	**2.6×10^–2^**
First AE	72	310	52 (69)	58 (78)	**2.16 (1.21 to** **3.87**)	**9.7×10^–3^**
First VTE	60	322	39 (75)	46 (79)	1.30 (0.78 to 2.17)	3.0×10^–1^
Onset of ESRD	14	245	43 (82)	64 (92)	**6.78 (1.78 to** **26.86**)	**6.5×10^–3^**
Overall mortality	50	379	66 (76)	66 (82)	**1.83 (1.02 to** **3.30**)	**4.3×10^–2^**

Patients in the extreme quartiles were included as affected individuals if they met the criteria for the examined manifestation; otherwise as censored individuals.

Values in bold indicate p<0.05.

*The mean age at the event includes only affected individuals.

†The mean survival is defined as the age at which 50% of individuals in each quartile are affected by the examined event.

‡Unadjusted.

AE, arterial event (myocardial infarction or ischaemic cerebrovascular disease); CVE, cardiovascular event (AE or VTE); ESRD, end-stage renal disease; GRS, genetic risk score; SDI, SLICC Damage Index^29^; VTE, venous thromboembolic event (deep vein thrombosis or pulmonary embolism).

Overall mortality increased with increasing GRS (p=9.4×10^–3^) (table 1) with the highest ORs for mortality observed in the groups of patients with the highest GRS (figure 3B). Patients in the high GRS-quartile further displayed a shorter mean survival compared with the low quartile (p=4.3×10^–2^) (table 2).

Because CVD is an important component of the SDI, we analysed survival until the first CVE separately. Patients in the high quartile displayed a decreased survival (p=2.6×10^–2^), with a mean age at the first event in affected individuals of 45 years, compared with 51 years in the low GRS-quartile (table 2). We subsequently divided CVE into arterial events (AE) and VTE and found that patients in the high GRS-quartile displayed a decreased survival until their first AE (p=9.7×10^–3^), but not their first VTE (p=3.0×10^–1^) (table 2).

**Figure 4:**
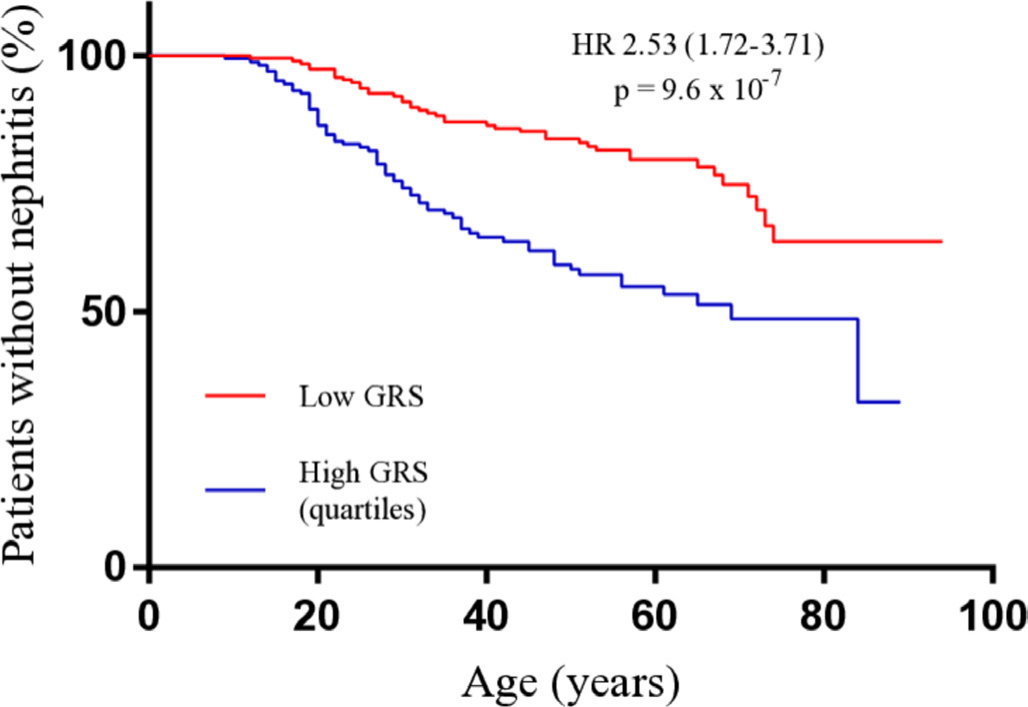
Survival comparison until nephritis onset in patients with a high or low GRS. Patients with a GRS in the extreme quartiles meeting the ACR-82 nephritis criterion, with a known date of nephritis diagnosis (n=109), were included as cases in the analysis, with their age at the time of nephritis diagnosis as the time variable. Patients in the extreme quartiles not meeting the nephritis criterion (n=245) were included as censored individuals, with their age at last-follow up as the time variable. The high and low quartiles were compared using the generalised Wilcoxon test. GRS, genetic risk score.

Analysis of the ACR–82 criteria^30^ showed that the prevalence of the renal and immunological criteria increased with increasing GRS (p=5.9×10^–5^ and p=3.6×10^–4^, respectively), with doubled odds of each manifestation in the high-to-low GRS-quartile comparison (table 1). In addition, dsDNA prevalence increased with increasing GRS (table 1). Patients in the high quartile further displayed a decreased mean survival until nephritis debut (p=9.6×10^–7^), with a mean age at nephritis onset of 31 years, compared with 39 years in the low GRS-quartile (figure 4). Next, we investigated the connection between cumulative genetics and renal dysfunction further. An increasing GRS was associated with higher stages of CKD and with development of ESRD, with five times elevated odds of ESRD in the high-to-low GRS-quartile comparison (p=1.0×10^–2^) (table 1). In addition, the mean survival until ESRD onset was decreased, with the mean onset in affected individuals occurring at 43 years in the high GRS-quartile, compared with 64 years in the low quartile (table 2). We subsequently analysed patients with positive renal biopsy results (n=222) and found that the prevalence of proliferative nephritis increased with increasing GRS (table 1).

Due to the relationship between a high GRS and an earlier onset of CVE, we investigated associations between the score and the prevalence of APS/anti-phospholipid antibodies (aPLs). The GRS was not significantly associated with APS; however, patients in the high GRS-quartile were more likely to have a positive aPL test (p=9.4×10^–3^), with more than doubled odds of being triple positive (table 1). Individually, lupus anticoagulant (LA), aβ_2_GP-I-IgG and aCL-IgG were significantly more prevalent in the high compared with the low quartile, with ORs of 2.12, 2.29 and 1.89, respectively (table 1).

To determine whether the association between a high GRS and early disease onset influenced other results, all previously significant associations were reanalysed with the age at SLE diagnosis included as an additional covariate. With the exception of the association between the GRS and proliferative nephritis on biopsy, all previously observed associations remained significant (online supplementary table 4).

Next, we calculated positive and negative predictive values (PPV and NPV) for our most important findings (online supplementary table 5). The GRS showed the highest predictive ability for ESRD, which at a GRS cut-off level of 9.5 had a specificity of 83%. At a prevalence of 11%,^32^ the PPV and NPV were 31% and 95%, respectively.

### Risk allele count, HLA-GRS and individual risk allele associations

To test whether the associations would remain when removing the weights of the GRS, all regression analyses were repeated using the unweighted RAC. With the exception of ESRD and the aPL variables, all associations remained significant (online supplementary table 6). We subsequently employed ROC curve analysis to compare prediction accuracies of the scores and found that the RAC generated a significantly better prediction of the immunological criterion^30^ whereas the GRS displayed a better prediction accuracy for ESRD, aβ_2_GP-I-IgG as well as presence of ≥3 aPLs (online supplementary table 6).

Next, we investigated associations between the HLA-GRS and clinical manifestations. With exception of negative associations with APS, aCL-IgM, aβ_2_GP-I-IgG and LA, no significant associations were found (online supplementary table 7).

Finally, all SNPs included in the GRS were analysed individually for association with the SDI. The *STAT4* (rs11889341) and *PRDM–ATG5* (rs6568431) risk variants were associated with increased SDI scores (OR 1.29 (1.10 to 1.52), p=2.9×10^–3^ and OR 1.31 (1.11 to 1.55), p=1.4×10^–3^, respectively) whereas *TMEM39A* (rs1132200) displayed an association with lower SDI scores (OR 0.70 (0.55 to 0.90), p=1.4×10^–3^).

## Discussion

Our study is the first to demonstrate an association between high cumulative genetic risk and survival, organ damage, cardiovascular disease, proliferative nephritis, ESRD and antiphospholipid antibodies in patients with SLE, introducing GRSs as a potential tool for prediction of disease severity. We employed both a weighted GRS and an unweighted RAC for our analyses, and their similar prediction accuracies regarding most outcomes—including organ damage and mortality—suggest that the added effect of multiple loci plays a more central role in the contribution to disease severity than the individual contribution by any high risk SNP.

The present study confers three important findings that may aid in explaining the association of the cumulative genetic risk with organ damage. First, we demonstrate that a high GRS is associated to an earlier onset of CVE, which is an important component of the SDI.^29^ Second, we found an association between a high GRS and presence of aPLs, including more than doubled odds of having a positive LA test. In addition to patients with aPLs having an increased risk of CVE,^33^ the LA test has been demonstrated to be the most predictive serological test for organ damage.^34^ Finally, the GRS was associated with renal involvement, higher stages of CKD, more severe biopsy classes including proliferative nephritis and, in particular, with ESRD. The renal domain is included as a separate item in the SDI, with ESRD generating more points than any other component of the index.^29^ Although these variables are likely contributors to our main result, there may be other important factors associated to both the GRS and to organ damage which were not examined in this study.

Our demonstration of a 6-year difference in SLE onset between the high and low GRS-quartiles supports previous findings by both Taylor *et al* 35 and Langefelt *et al*.^1^ A younger age at onset is associated with higher disease activity,^36^ an increased prevalence of nephritis and prolonged corticosteroid treatment,^37^ and the risk of acquiring organ damage in this group of patients is thus increased.^5 38^ We therefore included the age at SLE diagnosis as an additional covariate in our regression analysis and found only a small reduction in the effect size. Thus, the association between cumulative genetics and early disease onset may only to a limited extent explain our findings.

We found two individual variants positively associated with increased organ damage. The *STAT4* variant has previously been associated with a more severe disease phenotype including ischaemic stroke and increased SDI scores.^17–21^ Patients with SLE carrying this risk variant display an augmented IFN-γ production in T cells and elevated STAT1 expression in B cells.^39 40^ Because of the entailed potential therapeutic opportunity, we believe our confirmation of the association of this variant with organ damage is valuable. The *ATG5* gene encodes a protein involved in autophagy.^41^ Some studies have indicated that an altered function of this process increases the risk of lupus nephritis,^42^ which is in turn associated with damage accrual.

In analysis of the HLA-GRS, we found a negative association with aPLs and clinical APS. The reason for this may be that the DRB1*03:01 tag SNP rs1269852, due to its high prevalence and OR for SLE in our cohort, made a substantial contribution to the total score. Patients carrying this SLE-HLA allele are less likely to carry the DRB1*04 and *13 alleles, which are associated with secondary APS.^43^

The strength of our study is the large population including more than 1000 well-characterised patients with SLE, the comprehensive collection of clinical data and the long mean disease duration, allowing for long time follow-up of damage accrual. The validation of the GRS in a population including more than 15 000 patients and controls also confirms the significance of the cumulative genetic score. There are, however, some limitations. The retrospective approach of our study may confer a falsely low difference in overall survival between patients with high and low GRS, as only patients deceased after year 2000 are included in our study population. In addition, we lacked data regarding cumulative prednisolone dose and cumulative disease activity, which are important risk factors for the development of organ damage.^5 44 45^

Despite displaying moderate accuracy in the prediction of the examined manifestations, the combination of their relatively high prevalence, their severity and the benefit of early detection indicates a clinical relevance to the GRS. For example, an ESRD screening test with a GRS cut-off level of 9.5 would generate 22% positive samples, of which 31% would develop the complication compared with 5% of negative cases. Importantly however, the present study explores a GRS weighted by ORs for SLE rather than for renal manifestations. As there are several SNPs associated specifically with lupus nephritis,^46^ the method could be employed to design a nephritis-specific GRS with, plausibly, higher predictive accuracy.

In conclusion, a high GRS is associated with a more severe SLE phenotype involving an earlier onset of the disease, more organ damage and renal dysfunction, as well as impaired survival. Our results indicate that genetic profiling may provide a tool for predicting disease outcome and thus aid in the clinical decision process.
