# Supplementary material for: High genetic risk score is associated with early disease onset, damage accrual and decreased survival in systemic lupus erythematosus
Source: Ann Rheum Dis. 2019 Dec 11;79(3):363–9. doi: 10.1136/annrheumdis-2019-216227 (PMC7034364; doi:10.1136/annrheumdis-2019-216227)
Supplement: Supplementary data [file annrheumdis-2019-216227supp001.pdf]

Definitions

The age at nephritis diagnosis was defined as the age at which the patient first fulfilled the ACR-82 nephritis criterion.[1] The glomerular filtration rate (GFR) was calculated using the Modification of Diet in Renal Disease (MDRD) formula. Renal function was classified according to the chronic kidney disease (CKD) system.[2] The world health organization (WHO) 1995 classification system[3] was used for the classification of renal biopsies, where mesangial nephritis was defined as class I-II, proliferative nephritis as class III-IV, membranous nephritis as class V. Patients with biopsies displaying signs of nephritis but not meeting the criteria for any of the above classes,[3] were classified as *other*. Patients who had undergone renal transplantation or were receiving dialysis were considered to have end-stage renal disease (ESRD). dsDNA positivity was defined according to the ACR-82.[1]

A clinical diagnosis of APS was made based on thrombotic events and/or miscarriage in combination with positive tests for IgG aCL, IgG aß$_2$GPI or LA, and classified according to Miyakis *et al.*[4] Triple-positivity for aPL was defined as having positive tests for aCL (IgG or IgM) and aß$_2$GPI (IgG *or* IgM) and LA. Myocardial infarction (MI) required confirmation by electrocardiography and a rise in plasma creatine kinase, muscle and brain fraction (CK-MB) or troponine T, and ischemic cerebrovascular disease (ICVD) by computer tomography or magnetic resonance imaging. Venous thromboembolism (VTE) was defined as deep vein thrombosis, confirmed by venography or ultrasonography, and/or pulmonary embolism, confirmed by radionuclide lung scanning or angiogram. Cardiovascular events (CVE) was defined as any of the above events, whereas arterial events (AE) comprised MI and ICVD but not VTE.

Mortality data was collected at the end of follow-up for the patients in Uppsala, Linköing and Lund. Due to short follow-up of mortality for patients in Stockholm (Karolinska Institute), this information was updated, increasing the mean age at follow-up in these patients from 50 to 61 years. The Umeå patients were excluded due to short follow-up for mortality. In the mortality analysis, the mean age at follow-up for the whole study population was 57 years and the mean disease duration 21 years (n=776)

Genotyping and quality control

The discovery cohort were genotyped with the Illumina 200K Immunochip SNP array at the SNP&SEQ Technology Platform at Science for Life Laboratory in Uppsala, Sweden. Quality control was performed sample-wise and SNP-wise. Clustering and genotype calling was performed using Illumina's GenCall software. Sample call rate below 95% and SNPs with call rate below 100% were excluded. Samples with abnormal autosomal heterozygosity rate with more than 5SD from the mean of Wright's inbreeding coefficient F were excluded. A check for mislabelled gender was performed using Wright's inbreeding coefficient F, calculated from X chromosome data. Annotated females with F close to one or annotated males with F close to zero were excluded. Cryptic relatedness was analysed using KING software[5] and one sample from each related pair (up to 2nd degree of relatives) was excluded. Furthermore, principal component analysis (PCA) was performed on 1000 Genomes Project data and then used to project and exclude study samples falling more than 5SD from European populations in each of 5 principal components. SNPs with minor allele frequency (MAF) < 1% or with Hardy-Weinberg equilibrium (HWE) p-values within FDR < 5% (based on controls only) were excluded. In order to avoid any subject being assigned falsely low values for their cumulative

genetic load, only individuals with a 100% genotype success rate for all 57 SNPs were included in the study.

Construction of the genetic risk scores

Cumulative genetic risk scores (GRSs) were assigned to each individual based on SNPs with previous association with SLE at genome wide significance in the European population from the publication by Chen *et al*.[6] For SLE SNPs not included on the Immunochip, the SNP-proxy with the highest linkage disequilibrium (LD) (r2≥0.96) was selected. All SNPs were filtered for independent signals, removing the variant with the lowest SLE-odds ratio (OR) for SNPs in LD (r^2>0.2).The criteria described allowed for inclusion of 57 SNPs (supplementary table 2).

HLA-GRSs were assigned to each individual based on SNPs with previous association with SLE at genome wide significance in the European population from the publication by Chen *et al.[6]* All SNPs were filtered for independent signals, removing the variant with the lowest SLE-odds ratio (OR) for SNPs in LD (r^2>0.2). Due to high LD in the HLA region, this allowed for inclusion of 4 of 8 SNPs listed in Chen *et al,* see supplementary table 3. For each SNP, the natural logarithm of the OR for SLE susceptibility based on comparisons between the 1001 patients and 2802 controls in the discovery cohort, was multiplied by the number of risk alleles in each individual. The sum of all products for each patient was defined as the HLA-GRS.

The HLA-GRS followed a Gaussian distribution with higher mean scores in patients tha n in controls (mean (SD) 1.35 (0.78) compared to 0.95 (0.69)). In the discovery cohort, t

he probability that an individual had SLE was significantly higher in the highest, compa red to the lowest, HLA-GRS-quartile (OR 4.51 [3.58 –5.70], p=2.0×10^{-16}).

Statistical analysis

An ordinal regression was used to model the relationship between SDI scores and the cumulative genetic risk, with the outcome defined as a SDI score of 0, 1, 2, 3 or ≥4 points. This model was also used in analysis of CKD stages, with the outcome defined as stage 1, 2, 3, 4, or 5. Logistic regression was used for all analyses with binary outcomes, such as prevalence of each of the ACR-82 criteria. Age was included as a covariate in all analyses, and significant results were subsequently analysed in a second model, with the age at SLE diagnosis as an additional covariate. In the survival analyses, the generalized Wilcoxon test was employed to assess differences in survival between patients with a GRS in the high and low quartiles To compare the GRS and the RAC, we used receiver operating characteristics (ROC) curve analyses.

References

1. Tan EM, Cohen AS, Fries JF, et al. The 1982 revised criteria for the classification of systemic lupus erythematosus. *Arthritis Rheum* 1982;25:1271-7.

2. National Kidney F. K/DOQI clinical practice guidelines for chronic kidney disease: evaluation, classification, and stratification. *Am J Kidney Dis* 2002;39:S1-266.

3. Churg J, Bernstein J, Glassrock R. Renal disease: classification and atlas of glomerular diseases. 2 ed. New York: Igaku-Shoin.; 1995. 151-5 p.

4. Miyakis S, Lockshin MD, Atsumi T, et al. International consensus statement on an update of the classification criteria for definite antiphospholipid syndrome (APS). *J Thromb Haemost* 2006;4:295-306.doi:10.1111/j.1538-7836.2006.01753.x

5. Manichaikul A, Mychaleckyj JC, Rich SS, et al. Robust relationship inference in genome-wide association studies. *Bioinformatics* 2010;26:2867-73.doi:10.1093/bioinformatics/btq559

6. Chen L, Morris DL, Vyse TJ. Genetic advances in systemic lupus erythematosus: an update. *Curr Opin Rheumatol* 2017;29:423-33.doi:10.1097/BOR.0000000000000411
