# Supplementary material for: High genetic risk score is associated with early disease onset, damage accrual and decreased survival in systemic lupus erythematosus
Source: Ann Rheum Dis. 2019 Dec 11;79(3):363–9. doi: 10.1136/annrheumdis-2019-216227 (PMC7034364; doi:10.1136/annrheumdis-2019-216227)
Supplement: Supplementary data [file annrheumdis-2019-216227supp002.pdf]

Supplementary tables

Supplementary table 1. Prevalence of organ damage in the discovery cohort, per SDI domain and SDI score.

	Patients with ≥ 1 point in domain, n (%)	Total number of points in cohort
**SDI[1] damage domain**		
Ocular	181 (18)	196
Neuropsychiatric	209 (20)	260
Renal	106 (10)	166
Pulmonary	110 (11)	126
Cardiovascular	179 (17)	233
Peripheral vascular	150 (15)	163
Gastrointestinal	60 (6)	62
Musculoskeletal	252 (24)	324
Skin	142 (14)	156
Premature gonadal failure	39 (4)	39
Diabetes mellitus	53 (5)	53
Malignancy	102 (10)	114
Total (mean)	1 583 (1.58)	1 892 (1.89)
**Number of points on SDI[1]**	Patients, n (%)	
0	330 (33)	
1	231 (23)	
2	155 (15)	
3	123 (12)	
≥4	162 (16)	

SDI: Systemic Lupus International Collaborating Clinics/American College of Rheumatology damage index.[1]

Supplementary table 2. The 57 SLE susceptibility SNPs included in the Genetic Risk Score and the Risk Allele Count

Location	Lead SNP in publication[2]	Gene	SLE OR, Discovery cohort	Location	Lead SNP in publication[2]	Gene	SLE OR, Discovery cohort
1p13.2	rs2476601	*PTPN22*	1.30	6q23	rs2327832	*OLIG3-LOC100130476*	1.20
1q23.3	rs1801274	*FCGR2A*	1.11	7p15.1	rs849142	*JAZF1*	1.09
1q25.1	rs2205960	*TNFSF4*	1.34	7p12.2	rs4917014	*IKZF1*	1.28
1q25.3	rs17849502	*NCF2*	2.16	7q11.23	rs73366469	*GTF2IRD1-GTF2I*	1.73
1q25.3	rs10911363	*NCF2*	1.13	7q32.1	rs4728142	*TNPO3-IRF5*	1.55
1q31.3	rs34889541[a]	*CD45*	1.15	7q32.1	rs2070197	*TNPO3-IRF5*	1.86
1q32.1	rs3024505	*IL10*	1.34	8p23.1	rs2980512[a]	*FAM86B3P*	1.11
2p23.1	rs7579944	*LBH*	1.14	8p23.1	rs2736340	*BLK*	1.28
2p14	rs6740462	*SPRED2*	1.02	8q12	rs7829816	*LYN*	1.05
2q24.2	rs2111485	*IFIH1*	1.17	8q21	rs1966115	*PKIA-ZC2HC1A*	1.07
2q24.2	rs10930046	*IFIH1*	1.07	10q11.23	rs877819	*WDFY4*	1.03
2q32.3	rs11889341	*STAT4*	1.68	11p15.5	rs4963128	*IRF7-PHRF1*	1.31
3p14.3	rs6445972	*ABHD6-PXK*	1.06	11p13	rs2732552	*CD44*	1.16
3p14.3	rs6445975	*ABHD6-PXK*	1.17	11q13.1	rs1308020[a]	*RNASEH2C*	1.15
3q13.33	rs1132200	*TMEM39A*	1.27	11q13.4	rs3794060[a]	*DHCR7-NADSYN1*	1.02
3q25.33	rs564799	*IL12A*	1.20	11q24.3	rs7941765[a]	*ETS1-FLI1*	1.13
3q26.2	rs10936599	*MYNN*	1.04	12q24.12	rs10774625	*SH2B3-ATXN2*	1.18
				12q24.32	rs1059312	*SLC15A4*	1.30
4q24	rs10028805	*BANK1*	1.21	16p13	rs9652601	*CLEC16A-CIITA-SOCS1*	1.27
4q27	rs907715	*IL21*	1.06	16p11.2	rs34572943	*ITGAM-ITGAX*	1.51
5q31.1	rs7726414	*TCF7-SKP1*	1.01	16q13	rs223881	*CCL22*	1.16
5q33.1	rs7708392[a]	*TNIP1*	1.32	16q22.1	rs1170426[a]	*ZPF90*	1.17
5q33.3	rs2431697	*PTTG1-MIR146A*	1.22	16q24.1	rs2280381	*IRF8*	1.21
6p23	rs17603856	*ATXN1*	1.06	17q12	rs2941509	*IKZF3*	1.45
6p21.31	rs11755393	*UHRF1BP1*	1.27	17q25	rs930297	*GRB2*	1.00
6p21.31	rs2762340	*ANKS1A*	1.07	19p13	rs3093030	*ICAM1-ICAM4-ICAM5*	1.02
6q15	rs597325	*BACH2*	1.08	19p13	rs2304256	*TYK2*	1.36
6q21	rs6568431	*PRDM1-ATG5*	1.17	20q13.13	rs11697848	*RNF114*	1.02
6q23	rs6932056	*TNFAIP3*	1.83	22q11.21	rs7444	*UBE2L3-YDJC-HIC2*	1.22

SNP: single nucleotide polymorphism. We selected SNPs with a $p<5.0\times10^{-8}$ from Chen *et al.* [2] All SNPs have previously reported genome-wide levels of significance for SLE ($p<5x10^{-8}$). ORs for SLE development are based on comparisons between the 1001 patients and 2802 controls in the discovery cohort. [a]For SNPs not occurring on the Immunochip, the proxy SNP with the highest linkage disequilibrium[3] was used; for rs34889541, rs16843520 was used (r^2=1.00); for rs7708392, rs6889239 was used (r^2=0.99); for rs2980512, rs2948286 was used (r^2=0.96); for rs1308020, rs489574 was used (r^2=1.00); for rs3794060, rs4944062 was used (r^2=1.00); for rs7941765, rs6590343 was used (r^2=0.99); for rs1170426, rs1170427 was used (r^2=1.00).

Supplementary table 3. The 4 SLE susceptibility HLA-SNPs included in the HLA-Genetic Risk Score

Location	*Lead SNP in publication [2]*	*Gene*	*SLE OR, Discovery cohort*	*Location*	*Lead SNP in publication [2]*	*Gene*	*SLE OR, Discovery cohort*
6p21.32-33	rs3906272[a]	*HLA-C-HLA-B*	1.06	6p21.32-33	rs9270984[a]	*HLA-DRB1*	1.30
6p21.32-33	rs1269852	*MSH5-SAPCD1-TNXB-ATF6B*	2.59	6p21.32-33	rs2051549[a]	*HLA-DQA2*	1.74

SNPs selected from the publication by Chen *et al*. [2] SNP: single nucleotide polymorphism. All SNPs have previously reported genome-wide levels of significance for SLE ($p<5x10^{-8}$). [4]ORs for SLE development are based on comparisons between the 1001 patients in and 2802 controls in the discovery cohort. [a]For SNPs not occurring on the Immunochip, the proxy SNP with the highest linkage disequilibrium[3] was used; for rs3906272, s28732109 was used (r^2=1.00); for rs9270984, rs9270986 was used (r^2=1.00); for rs2051549, rs7453920 was used (r^2=1.00).

Supplementary table 4. Variables associated with the genetic risk score, adjusted for age at last follow-up and age at disease onset, in the Discovery cohort

		GRS, continuous	
	n (%)	*OR (95 % CI)[a]*	*P[b]*
Deceased at follow-up	130 (17)	**1.28 (1.05–1.57)**	**1.5×10^{-2}**
SDI scores[1]		**1.11 (1.03–1.22)**	**3.2×10^{-2}**
**SLE Criteria, ACR-82[5]**			
Photosensitivity	680 (68)	**0.88 (0.78–0.98)**	**2.2×10^{-2}**
Nephritis	342 (34)	**1.22 (1.09–1.37)**	**7.3×10^{-4}**
Immunological disorder	686 (69)	**1.24 (1.1–1.40)**	**3.6×10^{-4}**
dsDNA antibodies	477 (62)	**1.26 (1.11–1.44)**	**5.6×10^{-4}**
**Renal biopsy data[6]**			
WHO Class III-IV	133 (60)	1.16 (0.98–1.38)	8.1×10^{-2}
CKD stages[7]		**1.22 (1.05–1.42)**	**1.0×10^{-2}**
ESRD	24 (2)	**1.52 (1.06–2.19)**	**2.4×10^{-2}**
**Antiphospholipid antibodies**			
Any aPL	257 (38)	**1.14 (0.99–1.31)**	**6.6×10^{-2}**
Triple positivity[c]	119 (20)	**1.33 (1.11–1.61)**	**2.2×10^{-3}**
LA	121 (22)	**1.20 (1.00–1.44)**	**4.4×10^{-2}**
aCl-IgG	181 (27)	1.13 (0.97–1.31)	1.1×10^{-1}
aβ_2GP-I-IgG	19 (11)	**1.31 (1.10–1.56)**	**3.1×10^{-3}**

GRS: genetic risk score. OR: odds ratio. CI: Confidence interval. SDI: SLICC Damage Index.[1] WHO: World Health Organization.[6] CKD: chronic kidney disease.[7] ESRD: End-Stage Renal disease. LA: Lupus anticoagulant. aβ_2GP-I: anti-β_2 Glycoprotein-I. An ordinal (for SDI scores and CKD stages) or logistic (for all other outcomes) regression model was used. [a]OR for every increase of one point in the GRS (e.g. from 6.5 to 7.5). [b]Unadjusted. [c]Triple-positivity for aPL was defined as having positive tests for aCL (IgG *or* IgM) *and* aß$_2$GPI (IgG *or* IgM) *and* LA..

Supplementary table 5. Positive and negative predictive values for different cut-off levels of the genetic risk score (GRS).

	GRS cut-off[a]	Sensitivity	Specificity	PPV	NPV
**WHO class III-IV nephritis on biopsy[6]**					
	9,0	0,42	0,71	0,33	0,79
	9,5	0,32	0,83	0,39	0,79
	10,0	0,20	0,89	0,38	0,77
	10,5	0,11	0,94	0,38	0,76
**ESRD**					
	9,0	0,63	0,70	0,21	0,94
	9,5	0,63	0,83	0,31	0,95
	10,0	0,25	0,89	0,22	0,91
	10,5	0,13	0,94	0,21	0,90
**Nephritis[b]**					
	9,0	0,48	0,70	0,52	0,67
	9,5	0,34	0,83	0,57	0,65
	10,0	0,18	0,89	0,52	0,62
	10,5	0,10	0,94	0,53	0,61
**dsDNA antibodies[b]**					
	9,0	0,37	0,68	0,73	0,32
	9,5	0,24	0,80	0,74	0,31
	10,0	0,15	0,93	0,83	0,32
	10,5	0,09	0,97	0,88	0,31
**Any aPL[c]**					
	9,0	0,42	0,67	0,35	0,73
	9,5	0,27	0,78	0,34	0,71
	10,0	0,15	0,88	0,35	0,71
	10,5	0,08	0,93	0,33	0,70

GRS: genetic risk score. PPV: positive predictive value. NPV: negative predictive value. WHO: world health organization. ESRD: End-Stage Renal disease. Sensitivities and specificities were calculated for each clinical manifestation, for different cut-off levels of the GRS. As several of the variables are strongly related to age, patients ≤50 years of age were included in the analyses. [a]Patients with a GRS above and below the selected cut-off level were defined as positive and negative, respectively. [b]Defined according to ACR-82[5]. [c]Defined as having positive tests for aCL (IgG *or* IgM) *and/or* aβ_2GP-I (IgG *or* IgM) *and/or* LA.

Supplementary table 6. Comparison of the weighted genetic risk score (GRS) and the unweighted risk allele count (RAC) in the Discovery cohort

	Genetic Risk Score (GRS)		***Risk Allele Count (RAC)***		$p_{comparison}$[b]
	OR (95 % CI)[a]	*p*[c]	*OR (95 % CI)*[a]	*p*[c]	
Deceased at follow-up	**1.30 (1.07–1.59)**	**9.4×10^{-3}**	**1.08 (1.03–1.13)**	**3.4×10^{-3}**	5.8×10^{-1}
Male gender	1.07 (0.91–1.24)	4.2×10^{-1}	1.02 (0.98–1.06)	4.5×10^{-1}	5.7×10^{-1}
**SDI scores[1]**	**1.13 (1.03–1.24)**	**1.4×10^{-2}**	**1.03 (1.01–1.06)**	**1.2×10^{-2}**	-
**Extensive organ damage**	**1.16 (1.03–1.31)**	**1.7×10^{-2}**	**1.04 (1.01–1.07)**	**2.0×10^{-2}**	5.8×10^{-1}
**SLE Criteria, ACR-82[5]**					
Malar rash	0.94 (0.85–1.05)	2.6×10^{-1}	0.99 (0.96–1.01)	2.7×10^{-1}	5.7×10^{-1}
Discoid rash	0.94 (0.83–1.07)	3.4×10^{-1}	1.00 (0.97–1.03)	7.5×10^{-1}	7.1×10^{-1}
Photosensitivity	**0.88 (0.79–0.99)**	**2.6×10^{-2}**	**0.96 (0.94–0.99)**	**1.0×10^{-2}**	5.8×10^{-1}
Oral Ulcers	1.02 (0.91–1.15)	7.0×10^{-1}	0.99 (0.97–1.02)	7.0×10^{-1}	3.9×10^{-1}
Arthritis	0.91 (0.80–1.04)	1.5×10^{-1}	0.97 (0.95–1.01)	1.5×10^{-1}	2.9×10^{-1}
Serositis	0.95 (0.86–1.06)	3.6×10^{-1}	1.01 (0.98–1.03)	6.4×10^{-1}	1.1×10^{-1}
Nephritis	**1.29 (1.16–1.44)**	**7.0×10^{-6}**	**1.05 (1.02–1.09)**	**3.1×10^{-4}**	4.4×10^{-1}
Neurology	1.09 (0.92–1.29)	3.3×10^{-1}	1.01 (0.97–1.05)	6.3×10^{-1}	7.7×10^{-1}
Hematological disorder	1.05 (0.94–1.17)	3.7×10^{-1}	1.03 (1.00–1.05)	6.3×10^{-2}	6.8×10^{-2}
Immunological disorder	**1.29 (1.15–1.45)**	**1.6×10^{-5}**	**1.07 (1.04–1.10)**	**3.0×10^{-6}**	**4.7×10^{-2}**
dsDNA antibodies	**1.31 (1.15–1.50)**	**4.2×10^{-5}**	**1.08 (1.04–1.11)**	**7.0×10^{-6}**	5.2×10^{-2}
Sm antibodies	1.10 (0.90–1.33)	3.5×10^{-1}	1.02 (0.98–1.07)	3.2×10^{-1}	6.9×10^{-1}
ANA	1.37 (0.91–2.07)	1.4×10^{-1}	1.09 (0.99–1.20)	8.8×10^{-2}	3.3×10^{-1}
**Renal biopsy data[6]**					
WHO Class I-II	1.17 (0.86–1.59)	3.3×10^{-1}	1.04 (0.96–1.12)	3.5×10^{-1}	8.8×10^{-2}
WHO Class III-IV	**1.36 (1.14–1.62)**	**7.5×10^{-4}**	**1.07 (1.02–1.12)**	**3.3×10^{-3}**	2.0×10^{-1}
WHO Class V	1.10 (0.80–1.51)	5.6×10^{-1}	1.04 (0.96–1.12)	4.4×10^{-1}	3.3×10^{-1}
Other	1.01 (0.68–1.50)	9.5×10^{-1}	0.97 (0.88–1.07)	5.2×10^{-1}	4.6×10^{-1}
**CKD stages[7]**	**1.26 (1.09–1.47)**	**2.4×10^{-3}**	**1.04 (1.00–1.08)**	**3.3×10^{-2}**	-
**ESRD**	**1.65 (1.18–2.32)**	**3.6×10^{-3}**	1.08 (1.00–1.18)	6.0×10^{-2}	**3.5×10^{-2}**
**Antiphospholipid antibodies**					
Any aPL	**1.15 (1.00–1.32)**	**4.9×10^{-2}**	1.02 (0.98–1.05)	3.7×10^{-1}	1.2×10^{-1}
Triple positivity[d]	**1.30 (1.02–1.66)**	**3.2×10^{-2}**	1.03 (0.99–1.08)	1.4×10^{-1}	**9.9×10^{-3}**
LA	**1.21 (1.02–1.45)**	**3.3×10^{-2}**	1.02 (0.97–1.06)	5.0×10^{-1}	5.7×10^{-2}
aCl-IgG	1.14 (0.98–1.32)	9.1×10^{-2}	1.02 (0.99–1.06)	2.3×10^{-1}	2.6×10^{-1}
aCl-IgM	1.13 (0.91–1.41)	2.7×10^{-1}	1.02 (0.96–1.07)	5.5×10^{-1}	6.5×10^{-1}
aβ_2GP-I-IgG	**1.32 (1.11–1.58)**	**2.1×10^{-3}**	1.03 (0.99–1.08)	1.9×10^{-1}	**3.8×10^{-3}**
aβ_2GP-I-IgM	0.91 (0.61–1.35)	6.3×10^{-1}	0.94 (0.85–1.04)	2.4×10^{-1}	1.9×10^{-1}
Clinical APS	1.13 (0.96–1.34)	1.4×10^{-1}	1.03 (0.99–1.08)	1.3×10^{-1}	5.8×10^{-1}

GRS: genetic risk score. OR: odds ratio. CI: Confidence interval. SDI: SLICC Damage Index.[1] WHO: World Health Organization.[6] APS: antiphospholipid syndrome. CKD: chronic kidney disease.[7] ESRD: End-Stage Renal Disease. LA: Lupus anticoagulant. aβ_2GP-I: anti-β_2 Glycoprotein-I. A logistic regression model was used for all variables except SDI scores and CKD stages, which were analyzed using an ordinal regression model. Age at the last follow-up was included as a covariate in all analyses. [a]OR (95% CI) per unit increase in GRS or RAC. [b]p-values for comparisons of AUCs between the GRS and the RAC in receiver operating characteristics (ROC) analysis. Because the outcome was not binary, comparisons were not performed for SDI scores and CKD stages. [c]Unadjusterd. [d]Triple-positivity for aPL was defined as having positive tests for aCL (IgG *or* IgM) *and* aß$_2$GPI (IgG *or* IgM) *and* LA..

Supplementary table 7. Prevalence of clinical manifestations and serology versus associations with the HLA-GRS in the Discovery cohort

	HLA-GRS continuous *OR(95% CI)*	*P*[c]
Deceased at follow–up	0.95 (0.64–1.40)	0.79
Male gender	1.05 (0.83–1.34)	0.67
SDI scores[1]	0,87 (0.76–1.03)	0.074
**SLE Criteria. ACR–82[5]**		
Malar rash	0.95 (0.81–1.12)	0.55
Discoid rash	0.88 (0.73–1.07)	0.21
Photosensitivity	1.01 (0.85–1.20)	0.93
Oral Ulcers	0.96 (0.8–1.16)	0.68
Arthritis	1.01 (0.82–1.23)	0.95
Serositis	1.04 (0.88–1.22)	0.65
Nephritis	0.91 (0.76–1.08)	0.28
Neurology	0.86 (0.66–1.12)	0.27
Hematological disorder	1.17 (0.99–1.39)	0.065
Immunological disorder	0.91 (0.76–1.08)	0.28
dsDNA antibodies	0.98 (0.81–1.19)	0.87
ANA	1.00 (0.55–1.87)	0.99
**Renal biopsy data[6]**		
WHO Class I–II	0.88 (0.55–1.4)	0.58
WHO Class III–IV	0.9(0.69–1.16)	0.40
WHO Class V	0.96 (0.59–1.55)	0.86
Other[d]	1.09 (0.61–1.97)	0.76
ESRD	0.82 (0.47–1.38)	0.45
**Antiphospholipid antibodies**		
Any aPL	**0.77 (0.63–0.94)**	**0.012**
Trippel positive aPL[e]	**0.53 (0.36–0.77)**	**0.0012**
LA	**0.64 (0.48–0.84)**	**0.0015**
aCl–IgG	0.84 (0.67–1.05)	0.12
aCl–IgM	**0.65 (0.44–0.87)**	**0.0015**
aβ_2GP–I–IgG	**0.65 (0.49–0.84)**	**0.0015**
aβ_2GP–I–IgM	0.57 (0.28–1.08)	0.093
Clinical APS	**0.78 (0.6–1.00)**	**0.047**

HLA-GRS: HLA-genetic risk score. OR: odds ratio. CI: Confidence interval. SDI: SLICC Damage Index.[1] WHO: World Health Organization.[6] ESRD: End-Stage Renal disease. LA: Lupus anticoagulant. aCL: anti-cardiolipin. aβ_2GP-I: anti-β_2 Glycoprotein-I. APS: antiphospholipid syndrome. An ordinal (for SDI scores and CKD stages) or logistic (for all other outcomes) regression model was used.

All analyses were adjusted for age at last follow-up. [a]OR for the high compared to the low GRS-quartile. [b]OR for every increase of one point in the GRS (e.g. from 6.5 to 7.5). [c]Unadjusted. [d]Patients with biopsies displaying signs of nephritis but not meeting the criteria for any of the above classes,[6] were classified as other. [e]Triple positivity for aPL was defined as having positive tests for aCL (IgG or IgM) and a$ß_2$GP-I (IgG or IgM) and LA.

Supplementary figure legend

Supplementary figure 1. Validation of the Genetic Risk Score (GRS) in the replication cohort. A) The patients (n=5524) and healthy controls (n=9859) of the validation cohort were ordered according to their GRS and divided into 154 groups, each including 100 individuals (with exception of the last group, which consisted of 83 individuals). The prevalence of SLE in each group was subsequently plotted against the mean GRS. B) ROC curve analysis was employed to assess and compare the prediction abilities of the GRS and the RAC in the validation cohort (SLE n=5735, healthy controls n=10279).

References

1. Gladman D, Ginzler E, Goldsmith C, et al. The development and initial validation of the Systemic Lupus International Collaborating Clinics/American College of Rheumatology damage index for systemic lupus erythematosus. *Arthritis Rheum* 1996;39:363-9.

2. Chen L, Morris DL, Vyse TJ. Genetic advances in systemic lupus erythematosus: an update. *Curr Opin Rheumatol* 2017;29:423-33.doi:10.1097/BOR.0000000000000411

3. Machiela MJ, Chanock SJ. LDlink: a web-based application for exploring population-specific haplotype structure and linking correlated alleles of possible functional variants. *Bioinformatics* 2015;31:3555-7.doi:10.1093/bioinformatics/btv402

4. Bentham J, Morris DL, Graham DSC, et al. Genetic association analyses implicate aberrant regulation of innate and adaptive immunity genes in the pathogenesis of systemic lupus erythematosus. *Nat Genet* 2015;47:1457-64.doi:10.1038/ng.3434

5. Tan EM, Cohen AS, Fries JF, et al. The 1982 revised criteria for the classification of systemic lupus erythematosus. *Arthritis Rheum* 1982;25:1271-7.

6. Churg J, Bernstein J, Glassrock R. Renal disease: classification and atlas of glomerular diseases. 2 ed. New York: Igaku-Shoin.; 1995. 151-5 p.

7. National Kidney F. K/DOQI clinical practice guidelines for chronic kidney disease: evaluation, classification, and stratification. *Am J Kidney Dis* 2002;39:S1-266.
