# Supplementary figures and images for: High genetic risk score is associated with early disease onset, damage accrual and decreased survival in systemic lupus erythematosus
Source: Ann Rheum Dis. 2019 Dec 11;79(3):363–9. doi: 10.1136/annrheumdis-2019-216227 (PMC7034364; doi:10.1136/annrheumdis-2019-216227)

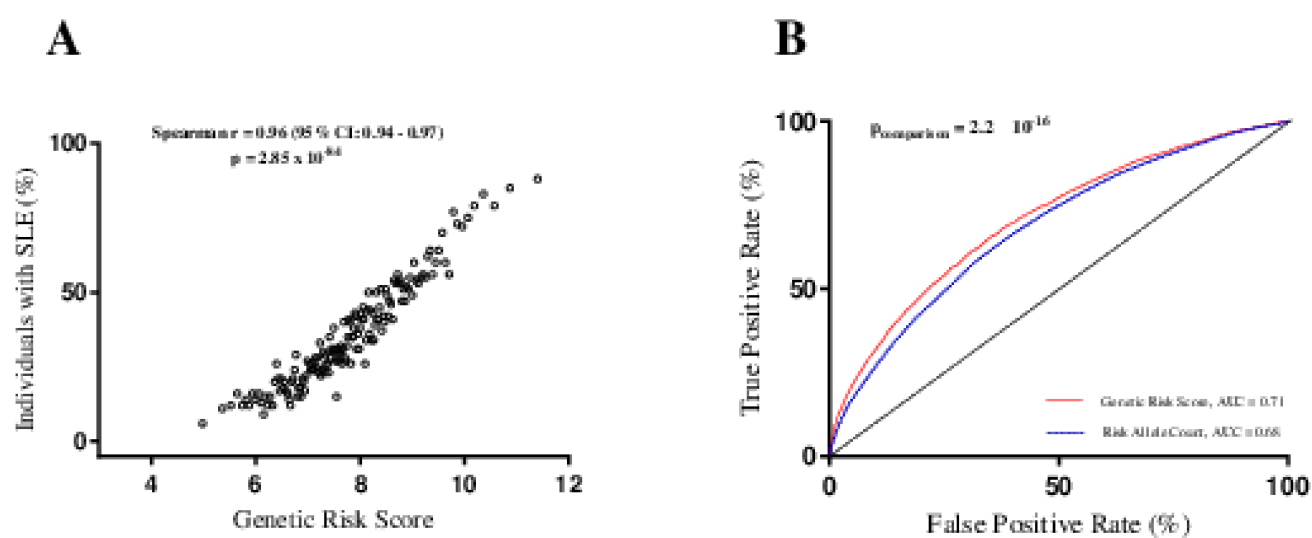

Supplement: Supplementary data [file annrheumdis-2019-216227supp003.pdf]
